# Classification of the height and flexibility of the medial longitudinal arch of the foot

**DOI:** 10.1186/1757-1146-5-3

**Published:** 2012-02-17

**Authors:** Mette Kjærgaard Nilsson, Rikke Friis, Maria Skjoldahl Michaelsen, Patrick Abildgaard Jakobsen, Rasmus Oestergaard Nielsen

**Affiliations:** 1 Clinic of Physical Therapy and Training, Ikast, Denmark; 2 Department of Orthopaedics, Thy- Mors Center of Head, Orthopaedics and Heart, Aalborg Hospital, Aalborg, Denmark; 3 Clinic of Physical Therapy and Training, Ulfborg, Denmark; 4 VIA University College, Holstebro, Denmark; 5 Orthopaedic Surgery Research Unit, The Head, Ortho and Heart Centre, Aarhus University Hospital, Aarhus, Denmark

**Keywords:** Medial longitudinal arch, Longitudinal arch angle, Navicular drop, Feiss line

## Abstract

**Background:**

The risk of developing injuries during standing work may vary between persons with different foot types. High arched and low arched feet, as well as rigid and flexible feet, are considered to have different injury profiles, while those with normal arches may sustain fewer injuries. However, the cut-off values for maximum values (subtalar position during weight-bearing) and range of motion (ROM) values (difference between subtalar neutral and subtalar resting position in a weight-bearing condition) for the medial longitudinal arch (MLA) are largely unknown. The purpose of this study was to identify cut-off values for maximum values and ROM of the MLA of the foot during static tests and to identify factors influencing foot posture.

**Methods:**

The participants consisted of 254 volunteers from Central and Northern Denmark (198 m/56 f; age 39.0 ± 11.7 years; BMI 27.3 ± 4.7 kg/m^2^). Navicular height (NH), longitudinal arch angle (LAA) and Feiss line (FL) were measured for either the left or the right foot in a subtalar neutral position and subtalar resting position. Maximum values and ROM were calculated for each test. The 95% and 68% prediction intervals were used as cut-off limits. Multiple regression analysis was used to detect influencing factors on foot posture.

**Results:**

The 68% cut-off values for maximum MLA values and MLA ROM for NH were 3.6 to 5.5 cm and 0.6 to 1.8 cm, respectively, without taking into account the influence of other variables. Normal maximum LAA values were between 131 and 152° and normal LAA ROM was between -1 and 13°. Normal maximum FL values were between -2.6 and -1.2 cm and normal FL ROM was between -0.1 and 0.9 cm. Results from the multivariate linear regression revealed an association between foot size with FL, LAA, and navicular drop.

**Conclusions:**

The cut-off values presented in this study can be used to categorize people performing standing work into groups of different foot arch types. The results of this study are important for investigating a possible link between arch height and arch movement and the development of injuries.

## Background

The structure and the movements of the foot arches are crucial for a person's wellbeing and for optimal function of the body [1]. Because the medial longitudinal arch (MLA) is the primary shock-absorbing structure of the foot, this area of the foot is particularly important for foot function [2]. To date, no firm conclusions can be made on the link between midfoot posture and the development of injuries. Finch [3] suggested conducting large scale prospective studies to investigate if the time to injury differs between individuals with different midfoot postures. In such a prospective cohort study, participants must be categorized into exposure groups based on their midfoot posture at baseline. Then, participants are followed over time to identify if the hazard of sustaining an injury varies among persons with different foot postures. However, to our knowledge no cut-off values have been presented to categorize participants into exposure groups based on their midfoot posture.

In general, two different approaches have been used to quantify midfoot characteristics. In the first approach, the maximum value is measured, which represents the maximal deformation of the MLA in a weight-bearing condition. In the second approach, the range of motion (ROM) is measured, which is the difference between the subtalar joint neutral position and the subtalar joint resting position measured in a weight-bearing position [4]. Different tests have been proposed to measure maximum values and ROM of the MLA. First, in 1909, the Feiss line (FL) was described [5], which classified foot type in the subtalar joint neutral and the subtalar joint resting positions. Second, navicular height (NH) and navicular drop (ND) were described by Brody in 1982 [6]. These methods allowed measurement, and evaluation, of the amount of pronation and its significance. Finally, in 1991 the longitudinal arch angle (LAA) was presented by Dahle et al. [7] to determine the relationship between foot type with subsequent knee pain or ankle sprains and to establish the interrater reliability of classifying foot type by visual observation. Because all three methods can be used to quantify the maximum value and ROM of MLA, cut-off values to categorize MLA into high arched, normal, and low arched should be provided for all three tests.

Previously, Williams et al. [8] used the standard deviation of the mean value to define cut-off values for the arch index of the midfoot, a fourth method used to quantify midfoot maximum values and ROM. Based on the cut-off values for the arch ratios, participants were divided into groups. Williams et al. [8] reported that a low or a high arch structure was associated with different injury patterns. In previous studies, participants have been categorized into exposure groups based on their alignment of other parts of the lower extremity [9]. Similarly, it is possible to categorize participants into exposure groups based on the static assessments of the NH [6], LAA [7], and FL [5]. However, to date the cut-off values for NH, LAA, and FL have only been reported as expert statements [6] or based on visual assessment [7]. To our knowledge, there is no report of using the prediction intervals to define cut-off values for NH, LAA, and FL. The primary purpose of this study was to identify cut-off values for maximum values and ROM values of NH, LAA, and FL based on the 68% and 95% prediction intervals.

While foot size and gender have been shown to be associated with measures of the midfoot in dynamic conditions, body mass index (BMI) or age do not appear to be associated with NH under dynamic measures [10]. However, to date it is unclear if these variables would be associated with foot posture in static measures. Furthermore, the total number of years of performing standing work and the number of hours worked at the time of measurement may be associated with the foot measures. The secondary purpose of this study was to determine the association between foot size, gender, BMI, age, years of performing standing work and hours worked at the time of measurement with NH, LAA, and FL, and if these parameters play a role to present a method to calculate cut-off values taking into account the association between different variables with NH, LAA, and FL.

## Methods

### Participants

Thirteen different companies in Jutland, Denmark, were contacted and asked if their employees who regularly perform standing work would participate in the study. Eight companies agreed to take part in the study, and their employees were asked to participate during their working hours. The sample population consisted of adult citizens from Central and Northern Denmark who were able to stand with their subtalar joint in neutral position. Informed written consent was obtained from each participant prior to the measurements. Overall, 258 men and women agreed to participate. Four participants were excluded because of data loss. Hence, data for 254 volunteers aged 18 to 68 years were included in the study.

### Procedures

The measurements were conducted by four clinicians. First, the clinician measured the participant's height, weight and foot length. A ruler (Folding rule, Probuilder, ABS Plastic) was used to measure foot length from the most posterior aspect of calcaneus to the tip of the longest toe. Then, the participant filled out a questionnaire about various clinical and demographic variables including age, gender, and injury history. Subsequently, the participant was placed standing barefoot on both feet with the subtalar joint in subtalar neutral position. Neutral position of the subtalar joint was defined as equal palpation of the medial and lateral aspects of the head of talus in relation to the navicular [10]. The following anatomical landmarks were identified by palpation and marked with a pen (CD/DVD marker, Relief); the centre of the medial malleolus, the navicular tuberosity, and the head of the first metatarsal bone. From these landmarks the maximum values of NH [6], LAA [11], and FL were measured for both feet with the subtalar joint in neutral position. Finally the participants were asked to relax, and NH, LAA, and FL were measured in the subtalar resting position. The differences between the measurements of NH, LAA, and FL with the subtalar joint in neutral position and measurements with the subtalar joint in the resting position were defined as ROM values for each test, respectively.

**Figure 1:**
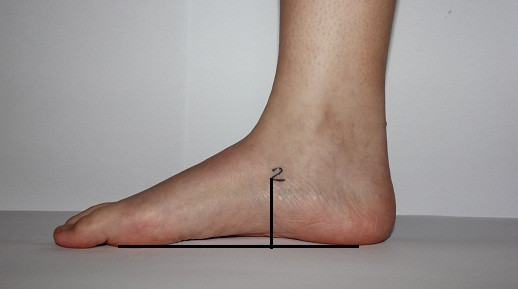
**Navicular height with the foot in subtalar neutral position**. The foot was in the subtalar neutral position and landmark 2 (navicular tuberosity) was used to measure the navicular height and the navicular drop. With the foot in a weightbearing position the measurements were repeated, and navicular tuberosity was marked again.

*NH: *The perpendicular distance between the floor and the navicular tuberosity was measured with a ruler (Figure 1). Sell et al. [12] reported an ICC ranging from 0.73 to 0.96 for the intertester and intratester reliability. Menz et al. [13] found NH to be significantly associated with the corresponding radiographic measure (Pearson r = 0.79).

**Figure 2:**
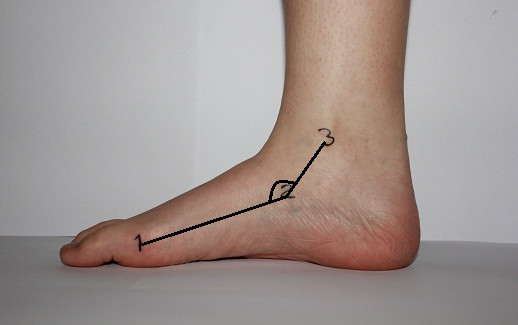
**Longitudinal arch angle with the foot in subtalar neutral position**. The foot was in its subtalar neutral position and the landmarks (1-3) were used to measure LAA. 1 head of first metatarsal bone; 2 navicular tuberosity; and 3 centre of the medial malleolus. A line was drawn from landmark 1 to 2 and from landmark 2 to 3. The superior angle between line 1 to 2 and line 2 to 3 was measured in degrees. With the foot in a weightbearing position, the measurements were repeated and the navicular tuberosity was marked again.

*LAA: *The centre of the goniometer (ProTerapi A/S) was placed at the navicular tuberosity, and the ends of the goniometer followed the landmarks on the centre of medial malleolus and the head of the first metatarsal. The angle (LAA) between the line from the medial malleolus to the navicular tuberosity and the line connecting the head of the first metatarsal bone and the navicular tuberosity was measured in degrees (Figure 2). Dahle et al. [7] reported a Kappa value for intertester reliability of 0.72 for visually assessing LAA, and Jonson et al. [14] reported an intratester and intertester ICC of 0.90 and 0.81, respectively.

**Figure 3:**
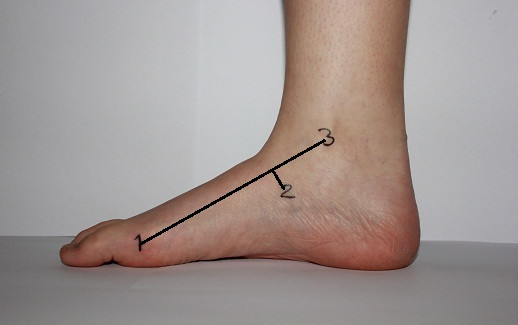
**Feiss line with the foot in subtalar neutral position**. The foot was in its subtalar neutral position and the landmarks (1-3) are used to measure Feiss line. 1 head of first metatarsal bone; 2 navicular tuberosity; and 3 centre of the medial malleolus. A line between landmark 1 and 3 was drawn and the perpendicular distance between landmark 2 and line 1 to 3 was measured. With the foot in a weightbearing position, the measurements were repeated, and the navicular tuberosity was marked again.

*FL: *A line was drawn between the centre of the medial malleolus and the head of the first metatarsal bone. Then, a ruler (15 cm, Relief) was used to measure the perpendicular distance in centimeters between the navicular tuberosity and the line from the medial malleolus to the head of the first metatarsal bone (Figure 3). If the navicular tuberosity was above the line, the measured distance was positive. If the navicular tuberosity was below the line, the value was negative. There are currently no studies showing the reliability of this test.

### Statistics

Significant differences between the right and left foot were found for LAA (*p *= 0.002) and FL (*p *= 0.035). However, these differences between right and left foot did not change the cut-off values significantly. Therefore, the differences were considered clinically insignificant. By pooling data from right and left foot, the assumption of independence is violated [15]. Therefore, data from either the right or the left foot was randomly included in the analysis. Descriptive data were presented as counts and percentage for dichotomous data, and as mean, standard deviation and 95% confidence interval for continuous data. All continuous data were normally distributed, tested by histograms and probability plots. Therefore, ± 1 standard deviation from the mean (68% prediction interval) were used as maximum value cut-off limits between normal and low arched and between normal and high arched feet. The ROM cut-off values between normal and flexible and between normal and rigid feet were calculated correspondingly. Furthermore, ± 2 standard deviations from the mean (95% prediction interval) were used as maximum value cut-off limits between low arched and severely low arched feet and between high arched and severely high arched feet. A corresponding approach was used for the ROM cut-off limits. Multivariate linear regression analysis was used to test the associated between age, BMI, foot length, years performing standing work and hours worked at the time of measurement with the measures of the medial longitudinal arch in male and female participants, respectively. The direction of the relationships was hypothesized to be positive for foot length and negative between NL, LAA, and FL and the covariates included in the analysis. Because no significant differences in LAA, FL, and NH measurements were found between healthy and previously injured participants, the data was not stratified based on injury status. All statistical tests were carried out in STATA (Texas, USA) version IC 11.0.

## Results

### Demographic characteristics

The demographic characteristics of the 254 participants of this study are presented in Table 1.

**Table 1:** Demographic characteristics

Variable	Mean	SD	95% CI	Range
Age (years)	39	11.7	37 to 40	18 to 64

BMI kg/m^2^	27.3	4.7	26.7 to 27.9	18.2 to 44.0

Sex*	198 m/56 f	N/A	N/A	N/A

Previous injuries during the last 12 months (yes/no)*	40/214	N/A	N/A	N/A

Standing work (years)	19	11.8	17.6 to 20.5	1 to 50

Standing work today (min)	259	114	244 to 273	0 to 720

Foot size (cm)	26.3	1.7	26.1 to 26.4	22 to 30.3

Demographic characteristics of the participants (n = 254). SD: standard deviation; 95% CI: 95% confidence intervals; cm: centimetre; min: minutes; * proportion presented. Standing work years represents the total number of years performing standing work. Standing work today represents the number of hours worked at the time of measurement. N/A = not available

Mean, standard deviation, 95% confidence interval and range for LAA, NH, and FL measurements are presented in Table 2.

**Table 2:** Mean, SD, 95% CI, and range of the test parameters

Maximum values n = 254				
**Test**	**Mean**	**SD**	**95% CI**	**Range**

NH (cm)	4.6	0.9	4.4 to 4.7	2.0 to 7.2

FL (cm)	-1.9	0.7	-2.0 to -1.8	-4.0 to 2.2

LAA (°)	142	10.4	141 to 143	114 to 172

**Range of motion values** **n = 254**				

**Test**	**Mean**	**SD**	**95% CI**	**Range**

ND (cm)	1.2	0.6	1.1 to 1.3	-0.3 to 3.2

FL (cm)	0.4	0.5	0.3 to 0.5	-3.8 to 2.3

LAA (°)	6	7	5 to 7	-15 to 26

Mean, standard deviation (SD), 95% confidence intervals (95% CI), and range between minimum and maximum values for navicular height (NH), navicular drop (ND), Feiss line (FL) and longitudinal arch angle (LAA). Maximum values represent the foot in its weight-bearing position. Range of motion represents the difference between subtalar neutral position and subtalar resting position with both feet in a weight bearing condition

Mean (± 1 SD) maximum values for NH, LAA, and FL were 4.6 cm (0.9 cm), 141.8 cm (10.4 cm), and -1.9° (0.7°), respectively. Without taking into account the effect of other variables, the 68% and 95% prediction limits for maximum values and ROM for ND, LAA, and FL are presented in Table 3.

**Table 3:** Cut-off values for NH, FL and LAA

Maximum values n = 254					
**Test**	**Severely low arch**	**Low arch**	**Normal**	**High arch**	**Severely high arch**

NH (cm)	< 2.7	2.7 to 3.5	3.6 to 5.5	5.6 to 6.4	> 6.4

FL (cm)	< -3.3	-3.3 to -2.5	-2.6 to -1.2	-1.3 to -0.4	> -0.4

LAA (°)	< 121	121 to 130	131 to 152	153 to 162	> 162

**Range of motion values** **n = 254**					

**Test**	**Very flexible**	**Flexible**	**Normal**	**Rigid**	**Very rigid**

ND (cm)	> 2.3	2.3 to 1.8	1.8 to 0.6	0.6 to 0.0	< 0.0

FL (cm)	> 1.3	1.3 to 0.9	0.9 to -0.1	-0.1 to -0.6	< -0.6

LAA (°)	> 19	19 to 13	13 to -1	-1 to -7	< -7

Cut-off values of the medial longitudinal arch maximum values and range of motion values without taking into account the association between the variables included in the regression models with NH, FL, and LAA. In relation to maximum values and range of motion values, the normal categories were defined by the 68% prediction limit cut-offs. Low arch and high arched feet as well as flexible and rigid feet were categorized based on the 95% prediction limit cut-offs. NH: navicular height; FL: Feiss line; LAA: Longitudinal arch angle

Normal maximum MLA and MLA ROM were for NH between 3.6 and 5.5 cm and between 0.6 and 1.8 cm, respectively. For LAA normal maximum values were between 131 and 152° and normal ROM values were between -1 and 13°. Normal maximum FL values were between -1.2 and -2.6 cm and normal FL ROM values were between -0.1 and 0.9 cm. These values represent the 68% prediction limits.

### Multivariate linear regression analysis

Results from the multivariate linear regression analysis testing the association between foot length, BMI, age, work performed on the day of testing, and number of year performing standing work with the different measures of MLA are presented in Table 4.

**Table 4:** Association between other variables with NH, FL, and LAA

	Women n = 56			Men n = 198		
	**B**	**SE**	***P***	**B**	**SE**	***P***

	**Navicular height (MV)**			**Navicular height (MV)**		

r^2^		0.06			0.04	

Intercept	3.93	2.35		4.31	1.43	

Foot length	0.04	0.10	0.703	-0.02	0.05	0.679

BMI	-0.012	0.02	0.556	0.01	0.02	0.501

Age	-0.02	0.02	0.169	-0.01	0.01	0.492

Work today	0.03	0.05	0.589	0.01	0.04	0.951

Year stand	0.01	0.01	0.357	-0.01	0.01	0.488

	**Navicular drop (ROM)**			**Navicular drop (ROM)**		

r^2^		0.13			0.04	

Intercept	0.14	1.52		-0.67	0.92	

Foot length	0.06	0.07	0.369	0.07	0.03	0.020

BMI	0.01	0.01	0.733	-0.01	0.01	0.528

Age	-0.01	0.01	0.789	-0.01	0.01	0.967

Work today	-0.06	0.03	0.060	-0.01	0.03	0.911

Year stand	-0.01	0.01	0.242	0.01	0.01	0.526

	**FL (MV)**			**FL (MV)**		

r^2^		0.17			0.09	

Intercept	1.13	1.85		1.83	1.14	

Foot length	-0.07	0.08	0.419	-0.11	0.04	0.005

BMI	-0.02	0.02	0.143	-0.01	0.01	0.729

Age	-0.02	0.01	0.051	-0.01	0.01	0.196

Work today	-0.01	0.04	0.766	-0.02	0.03	0.425

Year stand	0.01	0.01	0.273	0.00	0.01	0.806

	**FL (ROM)**			**FL (ROM)**		

r^2^		0.13			0.05	

Intercept	0.34	1.15		-0.12	0.78	

Foot length	-0.02	0.05	0.724	-0.01	0.03	0.843

BMI	0.01	0.01	0.296	0.01	0.01	0.051

Age	0.02	0.01	0.051	0.00	0.01	0.997

Work today	-0.01	0.03	0.582	0.03	0.02	0.195

Year stand	-0.02	0.01	0.035	-0.12	0.78	0.875

	**LAA (MV)**			**LAA (MV)**		

r^2^		0.12			0.11	

Intercept	161.10	29.50		183.30	15.50	

Foot length	-0.58	1.30	0.658	-1.20	0.54	0.026

BMI	-0.01	0.25	0.967	0.24	0.16	0.133

Age	-0.34	0.19	0.077	-0.34	0.13	0.008

Work today	0.87	0.63	0.177	-0.55	0.42	0.189

Year stand	0.21	0.18	0.243	0.09	0.12	0.462

	**LAA (ROM)**			**LAA (ROM)**		

r^2^		0.11			0.01	

Intercept	20.79	20.06		8.49	10.36	

Foot length	-0.61	0.88	0.494	-0.02	0.36	0.960

BMI	-0.05	0.17	0.774	-0.01	0.11	0.924

Age	0.21	0.13	0.102	-0.09	0.09	0.290

Work today	-0.17	0.43	0.699	0.06	0.28	0.817

Year stand	-0.30	0.12	0.018	-0.06	0.08	0.448

Multivariate linear regression analysis of the association between foot length (cm), BMI (kg/m^2^), age (years), hours worked at the time of measurement (work today), and total years with standing work (years standing) with maximum values (MV) and range of motion (ROM) in males and females, respectively. FL: Feiss line, LAA: Longitudinal arch angle. B coefficients with standard error were determined. *P *values < 0.05 were statistically significant for these analyses

Age only had a significant association with maximum LAA values among males. Among males, foot length was associated with ND and maximum LAA and FL. Work performed on the day of testing, BMI and number of years performing standing work had little or no association with midfoot measures. The regression equation used to calculate the normalized range for the tests taken into account other covariates was

$$\begin{array}{c} \text{Normal}\;\text{range}\left(\text{68}\%\;\text{prediction}\;\text{limits}\right)=(\text{Intercept}+\left({\text{B}}_{\text{foot}}*\text{foot}\;\text{length}\right)+\left({\text{B}}_{\text{BMI}}*\text{BMI}\right)+\\ \left({\text{B}}_{\text{Age}}*\text{Age}\right)+\left({\text{B}}_{\text{work testing day}}*\text{Work testing}\;\text{day}\right)+\left({\text{B}}_{\text{Year standing work}}*\text{Year}\;\text{standing}\;\text{work}\right)\pm \\ \text{Standard}\;\text{deviation}\left(\text{parameter}\right)), \end{array}$$

where work testing day was the number of hours of work performed on the day of testing and year standing work was the number of years performing standing work.

## Discussion

The primary purpose of this study was to identify cut-off values for maximum and ROM values of NH, LAA and FL based on the 68% and 95% prediction intervals. Cut-off values for maximum values or ROM in the static assessment of FL, NH, and LAA were presented without taking into account the effect of other variables. These cut-off values can be used by clinicians as a simple tool to categorize the MLA of persons who perform standing work. Furthermore, multivariate regression analysis was used to calculate cut-off values while taking into account foot size and other parameters.

Categorization of MLA can be calculated based on the regression equation and 68% and 95% cut-off values using the standard deviation reported in Table 2: cut - off value (regression equation) ± (1 or 2 standard deviations). For instance the 68% cut-off values/normal reference range for ND for a 30-year old male with a foot length of 28 cm, BMI of 25 kg/m^2 ^who has been working 4 hours at the time of measurement and worked 4 years performing standing work are:

(-0.67+(0.07*28)+(-0.01*25)+(-0.003*30)+(-0.003*4)+(0.005*4))±0.6=0.5cmto1.7cm.

This equation represents a precise method for calculating the cut-off values. This method can be used in future studies categorize participants into groups based on their midfoot posture.

The results of this study can be compared with other studies. Brody [6] reported normal amounts of ND of approximately 1 cm and considered a value of 1.5 cm as the upper boundary limit while no lower boundary limit was reported. In the current study, a normal ND was within the range of 0.6 to 1.8 cm, which corresponds well with the suggestions by Brody. However, it must be emphasized that the normal range of 0.6 to 1.8 cm was calculated without taking into account the effect of other variables.

To our knowledge, to date no cut-off values have been reported for NH. However, several studies [12,16-18] reported mean values of 3.7 to 4.7 cm, which is within the range of 3.6 to 5.5 cm used in the current study to categorize a normal foot.

Previously, LAA has been assessed visually by Dahle et al. [7]. Based on results from 55 participants, LAAs between 120 and 150° were classified as normal. The cut-off values of 131 to 152° reported in the current study were close to the proposed values reported by Dahle et al. [7]. However, cut-off values of 162 and 121° to distinguish between high arched and severely high arched and between low arched and severely low arched, respectively, differ considerably from those proposed by Dahle et al. [7] who suggested that participants with LAA close to 90° were classified as low arched, while participants with an LAA close to 180° were considered to have a high arch.

No studies were found which reported cut-off values or mean values for FL tests. Therefore, no external comparisons with the normal range of 0.9 to -0.1 cm can be made.

The results of the multivariate linear regression analysis revealed that age only had an association with maximum LAA values among males. However, the change in estimate per year was rather small. Therefore, the association between age with maximum LAA values is considered clinically insignificant. This result is in contrast to previous findings where a U-shaped pattern was reported between age and foot posture among children, in the general population and in the elderly [19]. However, because neither children nor elderly people were included in the current study, the insignificant association between age with most midfoot measures may be explained by the different age groups included in the two studies.

No clinically relevant and, in most cases, no statistically significant association between BMI, hours of standing work before the measurements, and total years performing standing work were found from with the different measurements of the MLA. These results are similar to those of Nielsen et al. [10] who found that age and BMI had no significant association with the foot position in dynamic conditions. However, in three studies [10,19,20], foot length was significantly associated with NH. In the current study, foot size had a significant association with most MLA parameters among males. Per 1 cm increase in foot size, ND was increased by 0.7 mm. For example, when comparing a 30 cm foot size with a 40 cm foot size, the expected increase in ND would be 0.7 cm. To avoid misclassification, such association between foot size and ND should be considered when categorizing subjects into high arched, normal and low arched groups, taking into account that a normal ND is between 0.6 and 1.8 cm. The effect of foot size can be accounted for by calculating the cut-off value based on the equation described above.

In the current study, NH, LAA, and FL were used to classify the midfoot posture in the sagittal plane. However, other tests for evaluating foot position have been described in the literature.

Recently, the Foot Posture Index (FPI) has been shown to be a valid and reliable tool for performing multiple segment, multiple plane evaluation of the foot as a whole [19,21,22]. Both the quantification of the midfoot measured in a single segment and the evaluation of the FPI in multiple segments can be used as tools to investigate the relationship between foot postures and injury development. One item of the FPI is the visual assessment of the arch height and congruence. Visual assessment of the MLA is a fast and simple alternative to describing midfoot posture compared with the tests presented in the current article. However, in their review, Razeghi and Batt [23] found one study where foot type classification based on direct observation demonstrated significantly higher variability. In another study, Cowan et al. [24] found the probability of two clinicians assessing the same foot as clearly flat ranged from 0.32 to 0.79, with a median probability of 0.57, while for clearly high-arched feet, comparable probabilities ranged from 0.00 to 1.00, with a median of 0.17. Based on these findings, it was concluded that there is a need for objective standards and quantitative methods for evaluating the MLA. The question arises whether or not the visual assessment of MLA is an appropriate tool in the FPI when valid and reliable alternatives such as NH or LAA exist.

Future studies should investigate if NH or LAA provide a better estimate of the midfoot than visual assessment of MLA which is used in the FPI. If this is the case, one may consider creating an extended FPI model where the visual assessment of the MLA would be replaced by NH or LAA. The method for calculating the cut-off values presented in the current study could be used to differentiate between the five categories (-2 to +2) currently used in the FPI.

Based on the cut-off values presented in this study, future studies can be conducted to investigate the injury incidence in persons with severely high, high, normal, low and severely low arched feet. If injury incidence varies between individuals with different foot postures, specific treatment modalities for modifying foot position and/or reducing the injury incidence would be warranted.

This study has some limitations. First, a relatively small sample of women was included in this study. Therefore, results of the regression analysis based on data from the 56 females should be interpreted with caution. Second, neutral position of the subtalar joint was defined as equal palpation of the medial and lateral aspects of the head of talus in relation to the navicular [10]. Previously, Pierrynowski et al. [25] investigated the proficiency of students and clinicians to place the foot in subtalar neutral. They found the rearfoot angle measured by experienced foot care specialists to be measured within +/- 3.0° of the subtalar neutral position 90% of the time. The corresponding value for the students was +/- 4.9°. Similarly, it has been stated that no method for measuring subtalar neutral position has been proven accurate and reproducible by different testers [26]. Therefore, the placement of the subtalar joint in its neutral position may be the greatest limitation in the current study. The ROM measurements presented may be interpreted with caution because the placement of the foot in subtalar neutral is part of the procedure for measuring the ROM.

## Conclusion

An approach to calculate cut-off values for NH, LAA, and FL measurements based on regression equations and standard deviation has been presented. These cut-off values can be used to differentiate between maximum foot arch values and foot arch ROM in the sagittal plane in people who regularly perform standing work. Based on these results, new prospective studies can be designed to elucidate if there is a link between arch height and arch movement and the development of injuries.

## Abbreviations

ROM: Range of motion; MLA: Medial longitudinal arch; BMI: Body mass index; NH: Navicular height; LAA: Longitudinal arch angle; FL: Feiss line; ND: Navicular drop; SD: Standard deviation; CI: Confidence intervals; Min: Minimum; Max: Maximum; cm: Centimeter; Min: Minutes; N/A: Not available; FPI: Foot Posture Index.

## Competing interests

### Financial competing interests

None of the authors hold any stocks or shares in an organization or have received reimbursements, fees, funding, or salary from an organization that may in any given way gain or lose financially from the publication of this manuscript, either now or in the future. None of the authors or organizations are currently applying for patents relating to the content of the manuscript.

### Non-financial competing interests

There are no political, personal, religious, ideological, academic, intellectual, commercial or any other competing interests related to the content of the manuscript.

## Authors' contributions

All authors read and approved the final manuscript. MKN carried out the acquisition of data and has been involved in drafting the manuscript. RF carried out the acquisition of data and has been involved in drafting the manuscript. MSM carried out the acquisition of data and has been involved in drafting the manuscript. PAJ carried out the acquisition of data and has been involved in drafting the manuscript. RON has made substantial contributions to conception, design, analysis, and interpretation of data as well as revised the manuscript critically for important intellectual content.
